# RASSF6; the Putative Tumor Suppressor of the RASSF Family

**DOI:** 10.3390/cancers7040899

**Published:** 2015-12-09

**Authors:** Hiroaki Iwasa, Xinliang Jiang, Yutaka Hata

**Affiliations:** 1 Department of Medical Biochemistry, Graduate School of Medical and Dental Sciences, Tokyo Medical and Dental University, Tokyo 113-8510, Japan; hiwammch@tmd.ac.jp; 2 Center for Brain Integration Research, Tokyo Medical and Dental University, Tokyo 113-8510, Japan; jiangxinliang.mbc@tmd.ac.jp

**Keywords:** apoptosis, cell cycle, Hippo pathway, MDM2, p53, Ras, tumor suppressor

## Abstract

Humans have 10 genes that belong to the Ras association (RA) domain family (RASSF). Among them, RASSF7 to RASSF10 have the RA domain in the *N*-terminal region and are called the N-RASSF proteins. In contradistinction to them, RASSF1 to RASSF6 are referred to as the C-RASSF proteins. The C-RASSF proteins have the RA domain in the middle region and the Salvador/RASSF/Hippo domain in the *C*-terminal region. RASSF6 additionally harbors the PSD-95/Discs large/ZO-1 (PDZ)-binding motif. Expression of RASSF6 is epigenetically suppressed in human cancers and is generally regarded as a tumor suppressor. RASSF6 induces caspase-dependent and -independent apoptosis. RASSF6 interacts with mammalian Ste20-like kinases (homologs of *Drosophila* Hippo) and cross-talks with the Hippo pathway. RASSF6 binds MDM2 and regulates p53 expression. The interactions with Ras and Modulator of apoptosis 1 (MOAP1) are also suggested by heterologous protein-protein interaction experiments. RASSF6 regulates apoptosis and cell cycle through these protein-protein interactions, and is implicated in the NF-κB and JNK signaling pathways. We summarize our current knowledge about RASSF6 and discuss what common and different properties RASSF6 and the other C-RASSF proteins have.

## 1. Introduction

Humans have 10 genes that are collectively called Ras association domain family (RASSF) [1,2,3]. Among them, six RASSF proteins, RASSF1 to RASSF6, have the Ras association (RA) domain in the middle region and the coiled-coil domain in the *C*-terminus. This *C*-terminal domain is named the Salvador/RASSF/Hippo (SARAH) domain, because Salvador and Hippo also have this domain. As described later, Salvador and Hippo are the founding members of the tumor suppressor Hippo pathway [4,5]. In contrast, the other four RASSF proteins, RASSF7 to RASSF10, have the RA domain in the *N*-terminal region and lack the SARAH domain [2]. Based on these differences, RASSF1 to RASSF6 are separately grouped as the C-RASSF proteins. The C-RASSF proteins have similar molecular structures and share the same interacting molecules. They are frequently silenced in human cancers and the low expression correlates with advanced stage and the poor prognosis [1,6]. Thus the C-RASSF proteins are generally considered to play a tumor-suppressing role. RASSF1A, one of the RASSF1 splicing variants, and Nore1 (RASSF5) are well studied and those studies are leading models in the research of the other C-RASSF proteins. Nevertheless, the C-RASSF proteins are still distinct from each other. Although all of them have the RA domain, their affinities for Ras proteins are different. RASSF1A is associated with the microtubules, while other C-RASSF proteins are not. RASSF1A and Nore1 harbor the C1 domain, while the other C-RASSF proteins do not. Only RASSF6 has the PDZ-binding motif. We focus on RASSF6 and discuss the similarities and the differences between RASSF6 and the other C-RASSF proteins.

## 2. RASSF6 and the PDZ Domain-Containing Proteins

**Figure 1:**
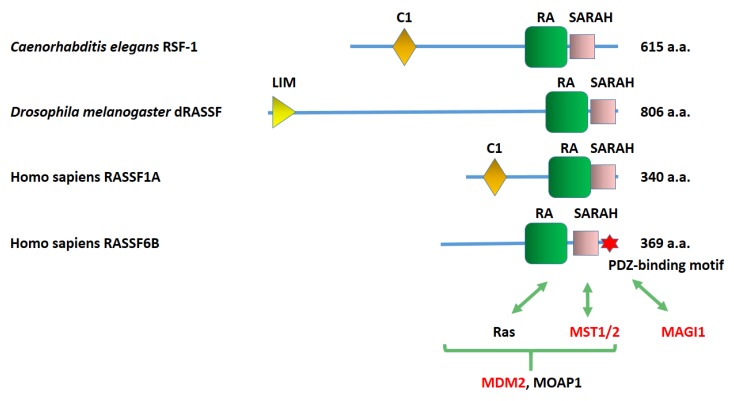
Structures of *Caernorhabditis elegans* RSF-1, *Drosophila melanogaster* dRASSF. *Homo sapiens* RASSF1A, and *Homo sapiens* RASSF6. C1, phorbol esters/diacylglycerol-binding domain. RA, Ras association domain. SARAH, Salvador/RASSF/Hippo domain. LIM, Zinc-binding domain present in Lin-11, Isl-1, Mec-3. The PDZ-binding motif of RASSF6 is depicted by a red star. The amino acid number of each protein is shown on the right. The RASSF6-interacting proteins are shown on the bottom. The interactions with MST1/2. MAGI1, and MDM2 are demonstrated at the endogenous level (red letters). Ras binds to the RA domain. MST1/2 (mammalian Ste20-like kinase 1/2) interacts with the SARAH domain. MAGI1 (membrane-associated guanylate kinase inverted 1) binds to the PDZ-binding motif. The interacting regions of MDM2 and MOAP1 (modulator apoptosis 1) are not precisely determined.

**Figure 2:**
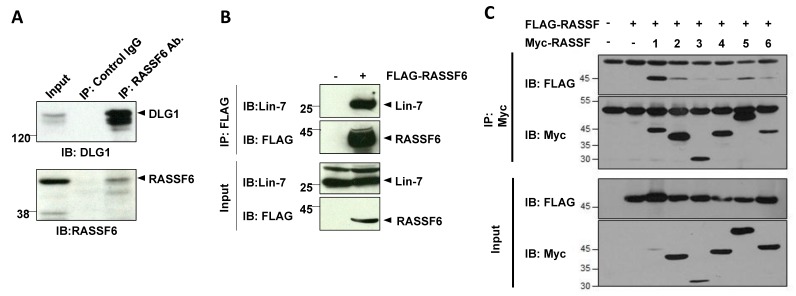
(**A**) Interaction of RASSF6 and DLG1. Endogenous RASSF6 was immunoprecipitated from rat liver with anti-RASSF6 antibody. DLG1 was co-immunoprecipitated; (**B**) FLAG-RASSF6 was expressed in HEK293 cells and immunoprecipitated with anti-FLAG antibody. Endogenous Lin-7 was co-immunoprecipitated; (**C**) FLAG-RASSF6 was co-expressed with Myc-RASSF1A, RASSF2, RASSF3, RASSF4, Nore1 (for simplicity, described as RASSF5 in this figure), and RASSF6. The immunoprecipitation was performed with anti-Myc antibody. The lower panel was the immunoblotting of the inputs. The upper panel was the immunoblotting of the immunoprecipitates. All Myc-C-RASSF proteins were co-immunoprecipitated with FLAG-RASSF6.

The human RASSF6 gene is encoded on chromosome 4. Four splicing variants are registered at the National Center Biotechnology Information database. RASSF6B is the longest isoform and contains 369 amino acids. RASSF6A, C, and D lack one or two exons. All RASSF6 variants have the PDZ-biding motif in the *C*-terminus (Figure 1). This is the prominent characteristic that distinguishes RASSF6 from the other C-RASSF proteins. We originally identified RASSF6 in the yeast two-hybrid screening by using membrane-associated guanylate kinase inverted 1 (MAGI1) as bait and confirmed that RASSF6 binds to the PDZ domain of MAGI1, although the physiological significance of this interaction is not yet clear [7]. In the reverse yeast two-hybrid screening using RASSF6 as the bait, we obtained not only MAGI1 but other PDZ domain-containing proteins (Discs Large Homolog 1/2/3, and Lin-7) (Figure 2). We have not deeply studied these interactions but there is a possibility that RASSF6 interacts with these PDZ domain-containing proteins.

## 3. The SARAH Domain-Mediating Interaction

The absence of good antibodies against RASSF6 and the low expression and solubility of endogenous RASSF6 significantly hamper the functional study on RASSF6-interacting molecules. Many interactions are predicted for RASSF6 and are demonstrated in the experiments by expressing heterologous proteins, but are lacking confirmation by endogenous interaction. Besides MAGI1, mammalian Ste20-like kinases (MST1/2) (mammalian homologs of Hippo) and MDM2 (an E3 ligase) are verified to interact with RASSF6 at the endogenous level [8,9] (Figure 1). MST1/2 have the SARAH domain, which interacts with the SARAH domain of RASSF6. The SARAH domain forms homo- and hetero-oligomers. As expected, the *in vitro* experiments demonstrate that RASSF6 interacts with the other C-RASSF proteins such as Nore1 via the SARAH domain (Figure 2). More interestingly, the SARAH domains of RASSF6 and WW45 (mammalian homolog of Salvador), both of which bind to the SARAH domain of MST1/2, do not interact with each other [8]. The interaction via the SARAH domain is important in the connection of RASSF6 to the Hippo pathway.

## 4. RASSF6 and the Hippo Pathway

**Figure 3:**
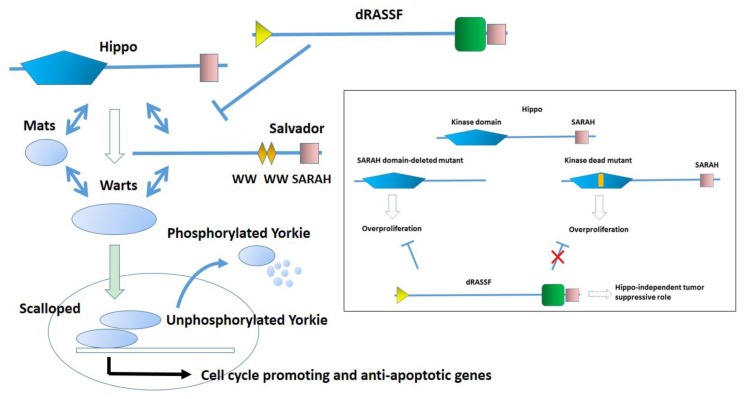
The core structure of the *Drosophila* Hippo pathway. Hippo and Warts form the kinase cassette. Mats and Salvador function as an activator and a linker to promote the Hippo-mediated activation of Warts. Salvador harbors two WW domains and the SARAH domain. Unphosphorylated Yorkie co-operates with Scalloped to regulate the transcription of cell cycle-promoting and anti-apoptotic genes. When Yorkie is phosphorylated by Warts, Yorkie is recruited from the nucleus to the cytoplasm and undergoes degradation (small entities symbolize degradation). dRASSF blocks the interaction between Hippo and Salvador. Inset: dRASSF suppresses the cell overproliferation phenotype caused by the *hippo* mutant lacking the SARAH domain but cannot rescue the phenotype caused by the kinase-dead mutant.

To discuss the relationship between RASSF6 and the Hippo pathway, we need to briefly overview the *Drosophila* Hippo pathway and *Drosophila* C-RASSF. The mosaic analysis to search for oncogenes in *Drosophila* revealed that the mutations of *hippo*, *salvador*, *mats*, and *warts* exhibit the similar cell overproliferation phenotype [4,5,10,11,12,13,14,15,16,17] (Figure 3). *Hippo* and *Warts* encode protein kinases. Hippo phosphorylates and activates Warts. Salvador and Mats function as adaptors and assist in the Hippo-mediated activation of Warts. That is, these four genes are not only genetically related but their products also physically interact with each other and form the signaling pathway that controls cell proliferation and apoptosis. This pathway was named the Hippo pathway. Thereafter, Yorkie was identified as a Warts-interacting protein [18]. Yorkie is a transcription co-activator and is phosphorylated by Warts. Unphosphorylated Yorkie co-operates with a transcriptional factor, Scalloped, and up-regulates cell cycle–promoting genes, while phosphorylated Yorkie is recruited from the nucleus to the cytoplasm and undergoes degradation. In the mutant animals with the loss-of-function of the Hippo pathway, Yorkie escapes the negative regulation and becomes constitutively active, resulting in the cell overproliferation. *Drosophila* has only one C-RASSF, dRASSF [19]. dRASSF depletion rescues the cell growth in the loss-of-function mutant of Ras1, suggesting that dRASSF antagonizes Ras1, although the underlying mechanism is unknown [19]. dRASSF also suppresses the cell overproliferation phenotype caused by the *hippo* mutant lacking the SARAH domain (Figure 3, the inset). These findings are consistent with the assumption that dRASSF is a tumor suppressor. Nevertheless, dRASSF competes with Salvador for the binding to Hippo and apparently blocks the activation of the Hippo pathway. Furthermore, dRASSF fails to rescue the phenotype caused by the kinase-dead *hippo* mutant (Figure 3, the inset).

There are several reports about the effect of the mammalian C-RASSF proteins on MST kinases (Table 1). The researchers focus on MST1 in some papers and study MST2 in others. The effects are evaluated in the *in vitro* kinase assay, in the *in vivo* autophosphorylation, in the stability, and in the down-stream activation of large tumor suppressor (LATS) kinases. In the *in vitro* kinase assay, MST kinases are co-expressed with C-RASSF proteins. The immunoprecipitated MST kinases are used on the beads or after the elution for the *in vitro kinase* assay. The co-expression of C-RASSF proteins tends to stabilize and increase MST kinases, which affects the results. Consequently, we detect some inconsistency among these papers.

**Table 1:** The effects of C-Ras-association domain family (RASSF) proteins on mammalian Ste20-like (MST) kinases. Experiment A; FLAG-MST1 was co-expressed with FLAG-RASSF1A or FLAG-Nore1 in HEK293 cells, immunoprecipitated with FLAG-antibody, and eluted with FLAG-peptide, and used for the *in vitro* kinase assay using myelin basic protein as a substrate. Experiment B; Myc-MST1 or Myc-MST2 was co-expressed with FLAG-RASSF1A, RASSF2, RASSF3, RASSF4, or RASSF6, immunoprecipitated with Myc- or FLAG-antibody, and used for the *in vitro* kinase assay using Mps one binder kinase activator 1 (MOB1) as a substrate. When anti-FLAG-antibody was used, the co-immunoprecipitated MST1 or MST2 was evaluated. Experiment C; FLAG-MST1 was co-expressed with Myc-RASSF2 in HEK293 cells, immunoprecipitated with FLAG-antibody, and used for the *in vitro* kinase assay using histone H2B as a substrate. Experiment D; FLAG-MST2 was co-expressed with Myc-RASSF6 or Myc-dRASSF in HEK293 cells, immunoprecipitated with FLAG-antibody, eluted with FLAG-peptide, and used for the *in vitro* kinase assay using MOB1 as a substrate.

C-RASSF	Effect on MST Kinases	Ref.
RASSF1A	Inhibition the autophosphorylation of MST1 in HEK293 cells.	[20]
	Inhibition of MST1 in the *in vitro* kinase assay (Experiment A).	[20]
	Dissociation of Raf-1 from MST2 and promotion of the interaction between MST2 and large tumor suppressor (LATS) 1.	[21]
	Activation of MST2 in the *in vitro* kinase assay (Experiment B).	[8]
RASSF2	Activation of MST1 in the *in vitro* kinase assay (Experiment C).	[22]
	Stabilization of MST2 in MCF-7 cells.	[23]
	Activation of MST2 in the *in vitro* kinase assay (Experiment B).	[8]
RASSF3	No significant effect on MST2 in the *in vitro* kinase assay (Experiment B).	[8]
RASSF4	Inhibition of MST2 in the *in vitro* kinase assay (Experiment B).	[8]
Nore1	Inhibition the autophosphorylation of MST1 in HEK293 cells.	[20]
	Inhibition of MST1 in the *in vitro* kinase assay (Experiment A).	[20]
	The co-expression of the active Ki-Ras activates MST1 in the complex of Nore1.	[20]
	Inhibition of the dimerization and the autoactivation of MST2.	[24]
RASSF6	Inhibition of MST1 and MST2 in the *in vitro* kinase assay (Experiment B and D).	[8]
	Inhibition of the autophosphorylation of MST2 in HEK293 cells.	[8]
dRASSF	Inhibition of MST2 in the *in vitro* kinase assay (Experiment D).	[8]
	Inhibition of the autophosphorylation of MST2 in HEK293 cells.	[8]

**Figure 4:**
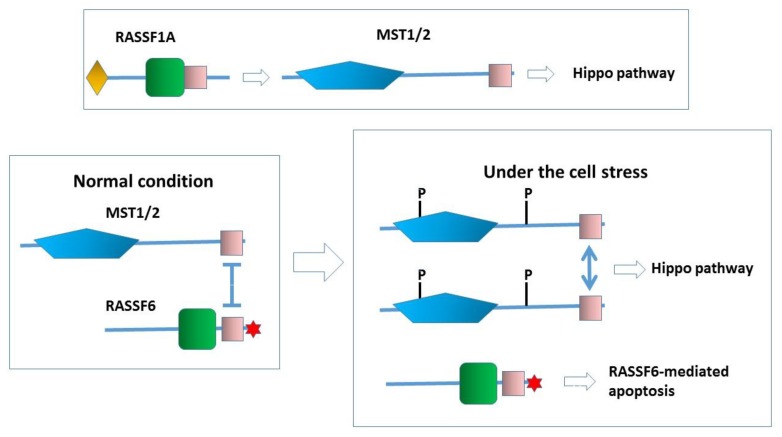
RASSF1A functions as an upstream activator for the Hippo pathway. For the precise mechanism of how RASSF1A activates MST2, the readers should refer to the other chapter in this issue. In contrast, RASSF6 works as a partner of MST kinases. Under the normal condition, RASSF6 and MST1/2 form a complex and inhibit each other. Under the condition that the Hippo pathway is activated, RASSF6 and MST1/2 are dissociated. MST1/2 are autophosphorylated (P). RASSF6-mediated apoptosis is concomitantly triggered. Note that, in the case that the machinery underlying RASSF6-mediated apoptosis is impaired, RASSF6 overexpression could lead to oncogenesis through the inhibition of the Hippo pathway.

The pioneering study about RASSF1A and Nore1 revealed the inhibitory effect on MST1 in the *in vitro* kinase assay and in the autophosphorylation *in vivo* [20]. The later study further demonstrated that Nore1 blocks the autoactivation of MST2 [24]. However, once MST2 is autoactivated, Nore1 does not inhibit MST2 anymore. At the cell level, Ki-Ras signaling recruits the complex of Nore1 and MST1 to the plasma membrane, and eventually activates MST1 [20]. RASSF1A releases MST2 from the inhibition by Raf-1 in cells to promote the interaction between MST2 and LATS1 and induce apoptosis [21]. RASSF2 activates MST1 in the *in vitro kinase* assay and stabilizes MST2 in cells [22,23]. In our *in vitro* kinase assay, RASSF1A activates MST2 [8]. Importantly, RASSF2, RASSF4, and RASSF6 inhibit MST2 under the same condition, and the chimera construct harboring the *N*-terminal region of RASSF1A and the *C*-terminal region of RASSF6 activates MST2, supporting that the activation of MST2 is specific for RASSF1A and is mediated by the *N*-terminal region of RASSF1A. Overall RASSF1A is likely to activate MST2 *in vivo* and the Hippo pathway (Figure 4, the upper part). It is understandable that the tumor-suppressive RASSF1A activates the Hippo pathway, but on the other hand, it raises a question about whether RASSF1A works in a different manner than dRASSF. On the other hand, the inhibition of MST kinases by RASSF6 is reminiscent of the inhibition by dRASSF of Hippo observed in *Drosophila*, but the mechanism may not be the same. dRASSF inhibits Hippo through blocking the binding of Salvador to Hippo, while RASSF6 and WW45 can simultaneously bind to MST1/2. RASSF6 is likely to block the homo-oligomerization of MST1/2 and inhibit the activation. As discussed below, RASSF6 is pro-apoptotic and its overexpression induces apoptosis in various cells [7,25]. However, RASSF6-induced apoptosis does not depend on MST1/2. On the contrary, MST1/2 significantly block RASSF6-induced apoptosis. At a glance, the tumor suppressor RASSF6 and the tumor suppressor Hippo pathway restrain each other. However, from another perspective, it is perceivable that RASSF6 and the Hippo pathway co-operate to induce apoptosis (Figure 4, the lower part).

When cells are exposed to stress such as the okadaic acid treatment, RASSF6 and MST1/2 are dissociated, resulting in the concomitant activation of RASSF6-mediated apoptosis and the activation of the Hippo pathway [8]. If this model is true for dRASSF and Hippo, the failure of dRASSF to suppress the phenotype of the kinase-dead *hippo* mutant can be explained. In the SARAH domain-lacking *hippo* mutant, dRASSF can trigger cell death because it is free of the inhibition by Hippo, but in the kinase-dead *hippo* mutant, dRASSF-mediated cell death is blocked. We are tempted to argue that RASSF6 is more similar to dRASSF than RASSF1A. However, more experimental evidence is required to support this notion. First, we need to know how dRASSF plays a tumor-suppressive role in the SARAH domain-lacking mutant. That mechanism should be independent of the Hippo pathway (Figure 2, the inset). As discussed below, MDM2 is important in the RASSF6-mediated apoptosis, but *Drosophila melanogaster* does not have the MDM2 orthologue. It is not clear how dRASSF antagonizes Ras1, either. Further studies are essential to conclude that RASSF6 and dRASSF function in a similar manner.

## 5. RASSF6, MDM2, and p53

**Figure 5:**
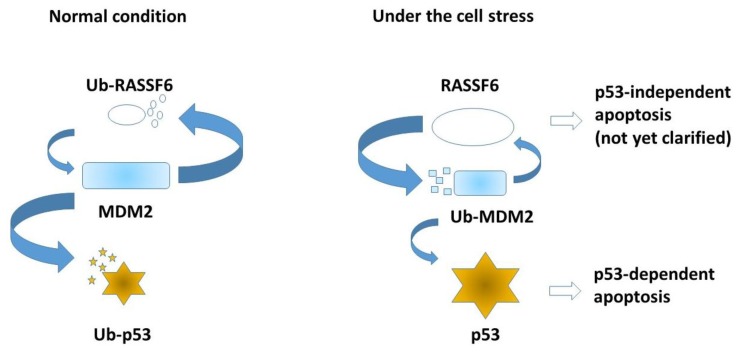
Under the normal condition, MDM2 degrades RASSF6 and p53. When cells are exposed to stress such as ultraviolet exposure, MDM2 self-ubiquitination is enhanced and p53 is stabilized. RASSF6 also induces apoptosis independently of p53, but the mechanism remains to be clarified. Small entities symbolize degradation of each ubiquitinated protein (Ub-RASSF6, Ub-p53, and Ub-MDM2).

If RASSF6 induces apoptosis and regulates the cell cycle independently of the Hippo pathway, how does RASSF6 function as a tumor suppressor? MDM2 is another important binding partner of RASSF6 [9]. The interaction between RASSF6 and MDM2 is confirmed through the co-immunoprecipitation of endogenous proteins. RASSF1A, RASSF3, and Nore1 are also shown to interact with MDM2 [26,27,28]. Thus, the interaction with MDM2 may be one of the common features shared by the C-RASSF proteins [26]. RASSF1A interacts with MDM2 and disrupts the MDM2-DAXX-HAUP interaction, and increases MDM2 self-ubiquitination. RASSF3 and RASSF6 also promote MDM2 self-ubiquitination, although the implication of DAXX-HAUP has not been tested [9,27]. Nore1 enhances the interaction between MDM2 and HIPK1 [28]. The mode of the interaction of RASSF6 with MDM2 has not yet been precisely determined, but the functional significance is obvious. When cells are exposed to the stress, RASSF6 promotes the self-ubiquitination and the subsequent degradation of MDM2, stabilizes p53, and causes apoptosis and G1/S arrest [9]. In the reverse experiment, the depletion of RASSF6 attenuates the ultraviolet exposure–induced up-regulation of p53. Based on these findings, we have proposed the model that, upon cell stress, MDM2, which destroys RASSF6 and p53 under the normal condition, starts to degrade itself in the RASSF6-dependent manner (Figure 5). RASSF3 works in the same manner to regulate apoptosis and cell cycle via MDM2-p53. There remain several questions to be answered. For instance, we do not know how RASSF6 is degraded and suppressed by MDM2 and simultaneously inhibits MST1/2. How MDM2 decides to adopt the self-ubiquitination and degradation fate is not clear, either.

## 6. RASSF6 and Other Pro-Apoptotic Molecules

RASSF6 induces apoptosis in caspase-dependent and caspase-independent pathways [7]. It induces Bax activation, cytochrome C release, and caspase-3 activation. It also causes the redistribution of apoptosis-inducing factor and endonuclease G. Although the importance of p53 for the tumor suppressor role of RASSF6 is unquestionable, RASSF6 can induce apoptosis even in p53-depleted cells [9]. This observation is important. It implies that RASSF6 may retain a tumor suppressor property in p53-compromised cells. The mechanism of the p53-independent RASSF6-induced apoptosis is not yet clear. After the report that RASSF1A binds modulator apoptosis 1 (MOAP1), the interaction between RASSF6 and MOAP1 was tested in the heterologous expression experiment and proposed as the mechanism underlying RASSF6-induced apoptosis [8,25,29,30]. MOAP1 knockdown attenuates RASSF6-induced apoptosis but does not reduce it in a p53-negative background, suggesting that MOAP1 functions in the same pathway as p53 [9]. In renal carcinoma cells, RASSF6 activates c-Jun *N*-terminal kinase (JNK) and JNK inhibitor suppresses RASSF6-induced apoptosis [31]. RASSF2 also activates JNK and induces apoptosis independently of the Hippo pathway [22]. Therefore, JNK signaling may be responsible for p53-independent C-RASSF-mediated apoptosis.

## 7. RASSF6 and Ras

Researchers concur on the point that Nore1 binds Ras proteins [32]. Endogenous Nore1 is co-immunoprecipitated with endogenous Ras, and the GTP-bound form of Ras specifically binds to Nore1 *in vitro*. However, whether all the other C-RASSF proteins directly bind Ras proteins has been a matter of debate. Here, we focus on RASSF6. When we prepared the cell lysates with the buffer containing 3 M urea to fully solubilize RASSF6 and used the lysates after dialysis for the immunoprecipitation, we could not detect the interaction of RASSF6 with Ras proteins, while Nore1 could bind Ras proteins under the same condition [7]. Allen *et al.* demonstrated that RASSF6 binds Ki-Ras in a GTP-dependent manner and that farnesylation is required [25]. In the comparative study using the bacterial recombinant protein, RASSF6 was shown to bind the dominant form of Ha-Ras [33]. According to these reports, we used a different lysis buffer without urea and detected the interaction between RASSF6 and Ras proteins. However, we have not yet succeeded in detecting the interaction between endogenous RASSF6 and Ras proteins. Nore1, when expressed alone in HeLa cells, is accumulated in the nucleus and the dominant active Ki-Ras remarkably recruits Nore1 to the plasma membrane, though it has no significant effect on the distribution of RASSF6. These findings indicate that RASSF6 may indeed interact with Ras proteins but with a much lower affinity than Nore1. What is the physiological significance of the interaction between RASSF6 and Ras? Nore1 interacts with MST1 in the presence of the dominant active Ras, and the SARAH domains of MST1 and Nore1 inhibit the Ras-induced apoptosis, suggesting that the interaction between MST1 and Nore1 is required for the Ras-induced apoptosis [34]. Nore1 depletion suppresses the Ras-induced senescence [35]. Together with the presence of the RA domain, these findings support that Nore1 functions downstream of Ras. In *C. elegans*, RSF-1 depletion blocks the multivulvar phenotype caused by the dominant active Ras mutant [36]. This observation further supports that the C-RASSF proteins are downstream targets of Ras. Although the effect of RASSF6 depletion on the Ras-induced apoptosis has not yet been tested, RASSF6 co-expression is demonstrated to enhance the Ras-induced apoptosis [25]. This finding suggests that RASSF6 may work to eradicate cells with Ras mutants and prevent oncogenesis. It is essential to determine whether and how Ras triggers RASSF6-mediated apoptosis.

## 8. RASSF6 and NF-κB (Nuclear Factor Kappa-Light-Chain-Enhancer of Activated B Cells) Signaling

The Nore1 deletion mutant without the RA domain and the SARAH domain can suppress the anchorage-independent cell growth of human lung cancer A549 cells, suggesting that Nore1 has a function independent of Ras and the Hippo pathway [37]. Likewise, RASSF6 is involved in apoptosis in various contexts, which may be independent of Ras and the Hippo pathway [7,8,25,38]. RASSF6 depletion suppresses tumor necrosis factor-α (TNF-α)-induced apoptosis in HeLa cells [7]. NF-kB is important in the TNF-α signaling. RASSF6 is shown to suppress the NF-κB-responsive reporter *in vitro* [25], but it remains to be clarified at the mechanistic level how RASSF6 plays a part in TNF-α-induced apoptosis and NF-κB signaling. RASSF6 is down-regulated in 3T3-L1 cells exposed to the conditioned medium of RAW264.7 cells containing TNF-α [39]. Once RASSF6 expression is suppressed, TNF-α signaling is enhanced and, as a consequence, RASSF6 is further down-regulated. This property implies that RASSF6 might be important in the pathophysiology of inflammatory diseases.

## 9. Conclusions

In the field of the C-RASSF protein studies, RASSF1A and Nore1 play leading roles. Other C-RASSF proteins have been studied after RASSF1A and Nore1. This is true for RASSF6, too. Many, including us, have compared RASSF1A and RASSF6 in their properties as a tumor suppressor, their ability to interact with MDM2, their involvement in TNF-α signaling and inflammation, as well as comparing them with Nore1- in the Ras-induced apoptosis. In most cases, the studies have revealed that RASSF6 behaves like RASSF1A and Nore1. However, RASSF6 is distinct from RASSF1A in the relationship with the Hippo pathway. Upon a certain cue, RASSF1A activates MST2 and triggers the Hippo pathway-dependent pro-apoptotic process (Figure 4, the upper part). RASSF1A serves as an upstream activator of the Hippo pathway. In contrast, RASSF6 can also work as a partner of Hippo in the Hippo pathway (Figure 4, the lower part). Which of RASSF1A and RASSF6 is more typical as the C-RASSF protein? RASSF1A is the best characterized C-RASSF protein, but it has exceptional properties. Most notably, RASSF1A is unique in the association with the microtubules [40,41,42]. The activation of MST2 through the competition with Raf1 may also be specific for RASSF1A [21]. Prior to our paper about RASSF6 [8], it was reported that Nore1 inhibits MST1 activation *in vivo*, and that the SARAH domain of Nore1 inhibits Ras-induced apoptosis [34]. The structural analysis revealed that the interaction in the heterodimer of Nore1 with MST1/2 is stronger than that in the homodimer of MST1/2 [43]. Consequently, MST1/2 preferentially form the complex with Nore1 and their autoactivation is inhibited [24]. In our hands, RASSF2 and RASSF4 also suppress MST2 activity *in vitro*, whereas RASSF1A activates it [8]. Interestingly, the chimera construct harboring the *N*-terminal region of RASSF1A and the C-terminal region of RASSF6 activates MST2 [8]. Song *et al.* reported that MST1 stabilizes RASSF2 and that RASSF2 activates MST1 *in vivo*, which is opposite to our *in vitro* observation [22]. However, as Cooper *et al.* showed that RASSF2 stabilizes MST2 [23], we speculate that RASSF2 also increases MST1 expression, which may explain the *in vivo* activation of MST1. RASSF3, like RASSF6, regulates p53 expression via MDM2. In alveolar rhabdomyosarcoma associated with Pax3-FOXO1, RASSF4 is highly expressed and leads to YAP1 activation by the suppression of the Hippo pathway [44]. As we have discussed about RASSF6 in the legend for Figure 4, if the machinery required for the Hippo-independent tumor-suppressive role of RASSF4 is deregulated, the oncogenic role of RASSF4 is understandable (Figure 4, the legend). These findings are in agreement with the model proposed for RASSF6 where the C-RASSF proteins form a complex with MST1/2 and inhibit the activation. We consider that RASSF1A is rather exceptional and RASSF6 is representative of the C-RASSF proteins.

## 10. Perspectives

To conclude that RASSF6 is a *bona fide* tumor suppressor, the experiments using RASSF6 knock out mice are necessary but the clinical data support the importance of RASSF6 in cancers. RASSF1A, RASSF2, RASSF4, and RASSF5 are reported to be silenced by high promoter methylation [6]. In contrast, it is discussed that no CpG is found in proximity to the first exon of RASSF6 and there is a possibility that RASSF6 expression is suppressed by an unknown mechanism other than DNA high methylation [6]. However, epigenetic suppression of RASSF6 is also reported for childhood leukemia, melanoma, and neuroblastoma cell lines [45,46,47]. The suppression of RASSF6 is demonstrated at the mRNA level in clear cell renal carcinoma, pancreatic cancer, gastric cancer and metastatic nasopharyngeal cancer cell lines [31,48,49,50]. The low expression of RASSF6 correlates with a poor prognosis. Experimentally, RASSF6 depletion enhances genomic instability [9]. Therefore, it is expected to improve the prognosis if we can recover RASSF6 expression. However, it is difficult to specifically reduce DNA methylation for a certain gene. DNA methyltransferase inhibitors, azacytidine and decitabine, are used as epigenetic modulators but their applications are limited due to cytotoxicity [51]. Instead of enhancing RASSF6 expression, we should discover a method to substitute for RASSF6-dependent apoptosis or cell cycle arrest. To reach this goal, we need to identify the mechanism of how RASSF6 fulfills its tumor suppressor role and to find out a drug target that surrogates RASSF6. As RASSF6 may be implicated in inflammatory diseases, the regulation of NF-κB signaling is also an important theme of RASSF6 research. Why humans have so many C-RASSF proteins is one of the naïve questions that we can raise. In *C. elegans*, RSF-1 is necessary for the multivulvar phenotype, which is observed by the hyperactivation of EGF-Ras signaling. We have to ask whether RASSF6 and other C-RASSF proteins are activated in response to the physiologically activated GTP-bound form of Ras and yield cellular consequences other than apoptosis and cell cycle arrest.
